# Supplementary material for: Causal Relationship between Aging and Anorexia Nervosa: A White-Matter-Microstructure-Mediated Mendelian Randomization Analysis
Source: Biomedicines. 2024 Aug 16;12(8):1874. doi: 10.3390/biomedicines12081874 (PMC11351342; doi:10.3390/biomedicines12081874)
Supplement: Supplementary file 1 [file biomedicines-12-01874-s001.zip › Supplementary Figure/Figure S9/Leaveoneout plot of MR analysis for ubm-b-1943 to AN.pdf]

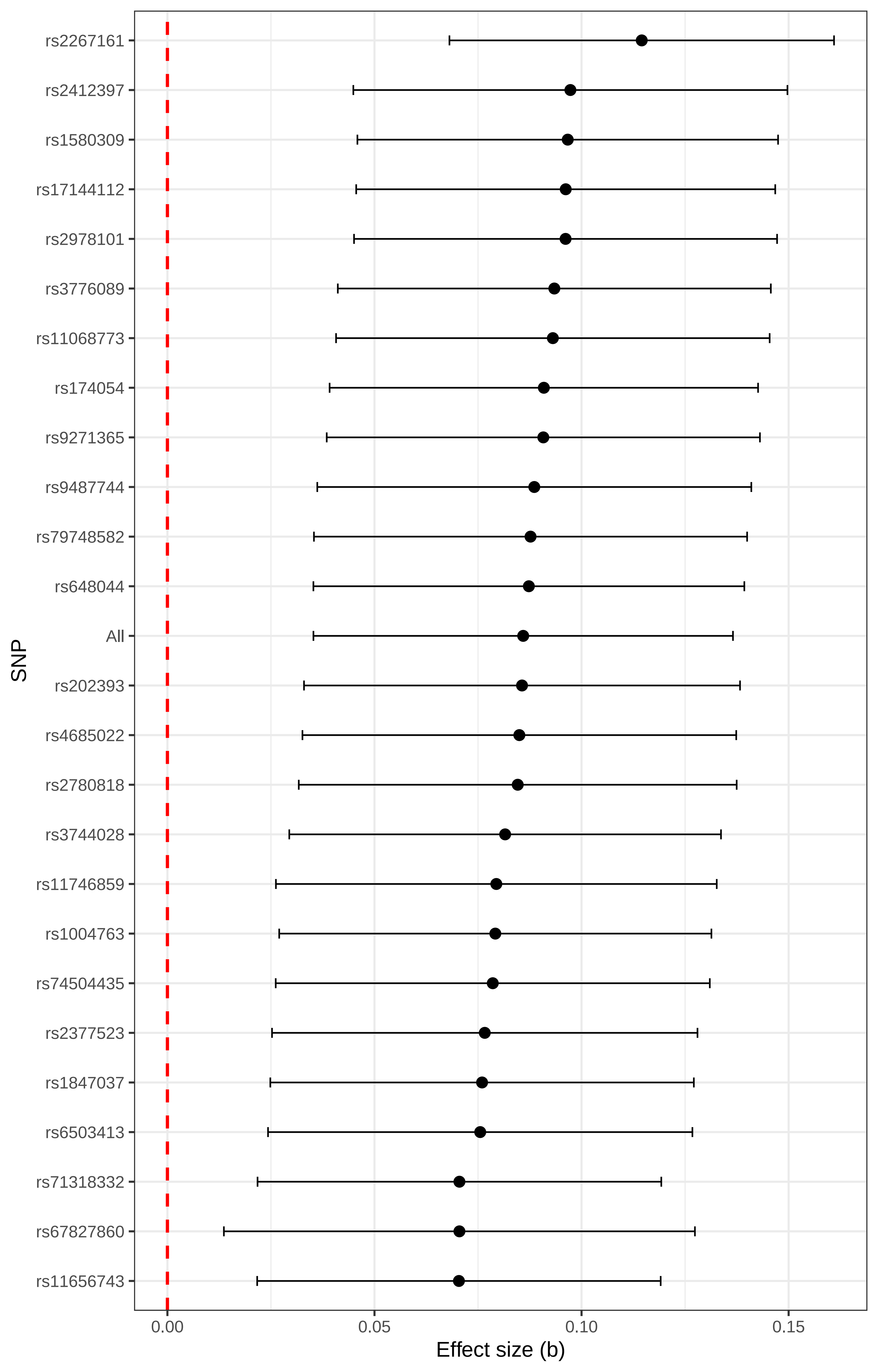

rs2267161
rs2412397
rs1580309
rs17144112
rs2978101
rs3776089
rs11068773
rs174054
rs9271365
rs9487744
rs79748582
rs648044
All
rs202393
rs4685022
rs2780818
rs3744028
rs11746859
rs1004763
rs74504435
rs2377523
rs1847037
rs6503413
rs71318332
rs67827860
rs11656743
SNP
0.00
0.05
0.10
0.15
Effect size (b)
